# Social-Ecological Predictors of Violent Firearm Use Among Youth

**DOI:** 10.3390/bs16101916

**Published:** 2026-10-16

**Authors:** Krista R. Mehari, Kelly O’Connor, Jasmine N. Coleman, Colleen S. Walsh, Lindiwe S. Mayinja, Phillip N. Smith

**Affiliations:** 1 Charles Stewart Mott Department of Public Health, College of Human Medicine, Michigan State University, East Lansing, MI 48824, USA; 2 Department of Surgery, Virginia Commonwealth University, Richmond, VA 23298, USA; kelly.oconnor@vcuhealth.org; 3 Department of Psychology, Virginia Commonwealth University, Richmond, VA 23298, USA; 4 Department of Psychology and Neuroscience, University of Tennessee-Knoxville, Knoxville, TN 37916, USA; jcolem71@utk.edu; 5 The Henry M. Jackson Foundation for the Advancement of Military Medicine, Inc., Bethesda, MD 20817, USA; colleen.walsh.ctr@usuhs.edu; 6 Department of Psychology and Human Development, Peabody College of Education and Human Development, Vanderbilt University, Nashville, TN 37235, USA; lindiwe.s.mayinja@vanderbilt.edu; 7 Department of Psychology, University of South Alabama, Mobile, AL 36688, USA; pnsmith@southalabama.edu

**Keywords:** guns, perpetration, peers, family, school, community

## Abstract

Firearm-related violent injuries are a leading cause of death among U.S. youth, yet little is known about what predicts youths’ violent use of firearms. This study examined how hypothesized predictors across social-ecological domains, identified through participatory research, explained violent firearm use. Data were collected from four public high schools in one Southeastern U.S. county. A total of 1126 students (*M_age_* = 16.17; 54.8% male; 50.9% Black, 33.6% White) completed the survey. Overall, 11.2% of participants reported having used a firearm against someone, and the model explained over 40% of the variance in violent firearm use, with 30 of the hypothesized 37 predictors significantly associated with violent firearm use. Notable factors included school-level poverty, firearm-related victimization, and violence in one’s social network (e.g., friend or family member injured or killed due to violence; friend committed a violent crime) and context (e.g., school safety problems; exposure to community violence). These findings underscore the importance of intervening in social networks and at the systems level in order to reduce the risk of youths engaging in violent firearm use.

## 1. Introduction

Homicide and assault are the leading causes of death among Black American youth, with over 90% of these deaths involving firearms (Centers for Disease Control and Prevention, n.d., 2021). However, little is known about what predicts youths’ violent firearm use,^1^ with most research predicting general weapons use, failing to differentiate victimization from violent firearm use, or focusing exclusively on individual level factors in high-risk samples (e.g., justice-involved youth; firearm-injured youth; Bender et al., 2021; Schmidt et al., 2019). There is an urgent need to identify social-ecological factors that are associated with youths’ violent firearm use to inform effective prevention strategies for reducing firearm-related injury and death.

Prior research has examined individual factors that predict violent firearm use among high-risk samples of youth. These factors include general and firearm-related victimization, a history of child physical abuse, witnessing community violence, delinquent behaviors (e.g., firearm carrying, alcohol and substance misuse), hopelessness, and beliefs supporting retaliatory violence (Erickson et al., 2006; Goldstick et al., 2019; Lee et al., 2022; McGee et al., 2017; Osborne et al., 2024; Pardini et al., 2021; Rowan et al., 2019; Stoddard et al., 2011; Sumner et al., 2016). Social-ecological theory, however, highlights the need to understand the development of youths’ behaviors within the context of social and built environments (Bronfenbrenner, 1977). As predicted by social-ecological theory, population-level research has identified that firearm-related violence both clusters in place and spreads through social networks (contagion and diffusion effects; Green et al., 2017; Loeffler & Flaxman, 2018; Papachristos et al., 2015; Towers et al., 2015). These findings underscore the need to understand what social-ecological factors specifically predict an individual’s violent use of firearms.

Compared to research on individual-level predictors, research on factors from other social-ecological domains (e.g., community factors such as school or neighborhood characteristics) or social-cultural factors is severely limited, with most contextual research focusing on families and peers. For example, family predictors include domestic or family violence and firearm access in the home (Huesmann et al., 2021; Ruback et al., 2011; Sumner et al., 2016). In the peer domain, peer delinquent behaviors, including friends’ firearm carrying and gang membership, predicts youths’ violent firearm use (Pardini et al., 2021). The limited research examining neighborhoods as predictors of individual-level violent firearm use has identified neighborhood violence as a risk factor for violent firearm use and adult social support as a promotive factor (Huesmann et al., 2021; Osborne et al., 2024; Pardini et al., 2021). Similarly, population-level research has identified community factors associated with community-level firearm violence, including segregation, income and poverty, employment, housing vacancy, and heat index (Goin et al., 2018; Tracy et al., 2019). In the cultural domain, exposure to violent media predicts youths’ violent firearm use (Huesmann et al., 2021). Similarly, population-level research has found that violence in popular television predicts increases in national firearm-related homicides (Jamieson & Romer, 2021).

Despite their profound impact, few studies have systematically examined the influence of factors across multiple social-ecological domains. Examining a breadth of potential predictors provides a greater understanding of how these factors across social-ecological domains are uniquely associated with violent firearm use. Furthermore, identifying contextual factors that predict violent firearm use is consistent with a large body of research that shows that these factors hold promise as intervention targets due to their broader scope of influence, population-level impact, and focus on root causes of violent firearm use.

The purpose of this study was to use participatory research to identify a range of possible predictors of firearm-related violence and to examine the extent to which those factors explained variance in youths’ violent use of firearms. In a participatory process, we selected social-ecological factors across intra-individual, peer, family, school, neighborhood, and cultural domains that were emphasized by members of communities impacted by violence. We hypothesized that all the selected factors across domains would be associated with youths’ violent use of firearms. Analyses about what factors predicted unique variance within and across domains were exploratory.

## 2. Materials and Methods

### 2.1. Participants

**Figure 1:**
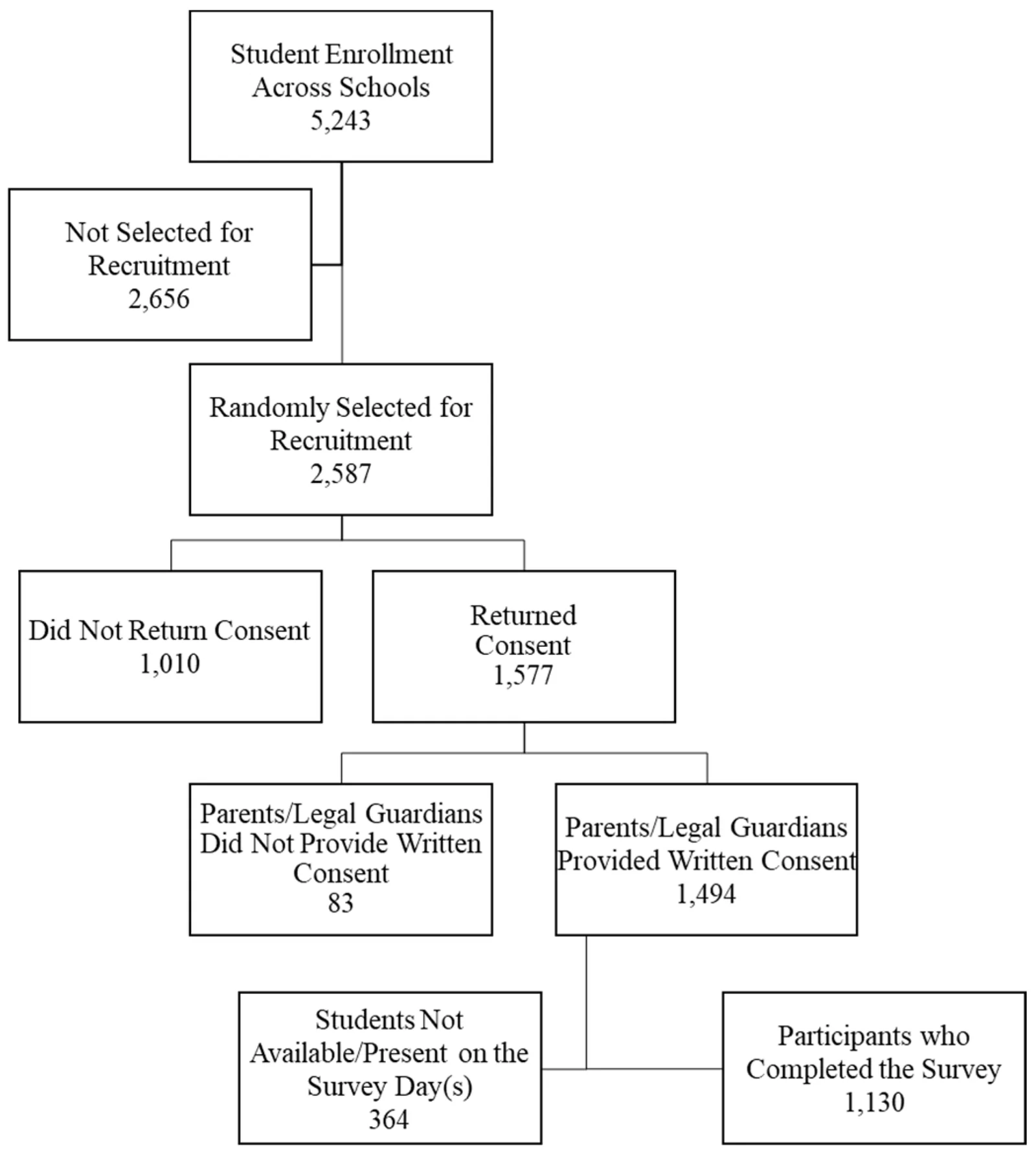
Flow Chart of Participant Recruitment.

Data for this study were collected in one Southeastern U.S. county. Four high schools from two public school districts were recruited to establish a sample reflective of the county’s demographics concerning urbanicity, income, and race/ethnicity. Two schools were categorized as high poverty, with over 85% of students eligible for free or reduced-price lunch (FRPL); the others were categorized as mid-low poverty (<50% qualifying for FRPL) (National Center for Education Statistics, n.d.). Two schools were in urban settings and two in suburban settings. Youth were eligible if they were at least 14 years old, and if they attended classrooms randomly selected for recruitment. The recruitment goal was 300 students per school (total *n* = 1200). Of the 2587 students who were enrolled in the randomly selected classrooms, 60.9% (*n* = 1577) returned consent forms, and 94.7% (*n* = 1494) received written consent. Ultimately, 1130 students were present during data collection and completed the survey, and 1126 provided usable data, creating the analytic sample (see descriptives in Table 1, and see Figure 1 for recruitment flow chart). Within the analytic sample, 61% of students attended a high-poverty school. Participants were ages 14 to 21 years, with an average age of 16.17 years old (*SD* = 1.15). The sample was 55% male and 45% female. Participants identified as Black (51%), White (34%), or another race (16%). Eighteen percent of participants identified as a sexual or gender minority.

### 2.2. Participatory Research

Our measures and procedures were developed through a participatory process guided by three advisory boards (firearm owners, young adults in high-violence areas, and community leaders working with youth in high-violence areas). Community leaders included school personnel (teachers, principals); clergy; business owners; leaders of afterschool and summer sites; police officers and leaders; and organizers. Members of the research team (community research assistants with relevant lived experience as firearm owners or living in high-violence areas; Smith et al., 2025) provided day-to-day participatory input and feedback. In the beginning of the process, advisory boards and community research assistants brainstormed factors within social-ecological domains (e.g., family, schools) that they believed predicted violence. These hypotheses shaped the development of a qualitative interview protocol that was then administered to 250 individuals with lived experience in high-violence areas or as firearm owners (for more details, see Mehari et al., 2026b). The emerging themes from the interviews provided additional hypothetical predictors. The research team then examined the literature for measures to assess the constructs of interest. When the team could not find existing evidence-based measures or when the existing measures did not capture the full range of the proposed construct, we created or adapted measures as needed, also using a participatory process. Specifically, items were generated from qualitative interviews and advisory board discussions; the research team refined and prioritized items through voting; and advisory boards participated in cognitive testing and recommended revisions for language and content (for a comprehensive discussion of the participatory measurement development process, see Mehari et al., 2026a).

### 2.3. Survey Procedures

All study procedures were approved by the university’s Institutional Review Board, school district leaders, and school administrators. Data were collected in December 2022, and in February, March, and May 2023. The research team visited the schools to share study information and provide parental consent forms to eligible students. Participants received $10 upon returning a signed consent form, regardless of whether they agreed to participate. Surveys were completely anonymous and were protected by a Certificate of Confidentiality. Typically, data collection spanned two days at each school, and surveys administered electronically through Qualtrics, with research team members present to address questions. On average, surveys lasted approximately 30 min. Participants received $25 for completing any portion of the survey.

### 2.4. Measures

**Cultural domain**. Participants completed a six-item measure of past year media exposure to violence and crime (1 = *Never* to 5 = *Almost every day*). One item came from a prior measure (Intravia et al., 2017) and five were generated through participatory action research (e.g., “People I know have posted photos of themselves with guns online;” α = 76; Mehari et al., 2025b).

**Community Domain.** Participants rated four items to indicate their past six-month frequency of witnessing community violence (1 = *Never* to 6 = *20 or more times*, e.g., “Seen someone getting beat up or mugged?”, α = 0.76) (Richters & Saltzman, 1990). An adapted version of the Neighborhood Collective Efficacy Scale (Sampson et al., 1997) was used to assess safety and informal social control (e.g., “This is a friendly neighborhood”, α = 0.72); two items assessed neighbors’ likelihood to intervene from 1 (*Very unlikely*) to 5 (*Very likely*), and four assessed safety from 1 (*Strongly disagree*) to 5 (*Strongly agree*). Two subscales measured presence of a caring adult (1 = *NO!* to 4 = *YES!*): Positive (five items, “There are adults I can count on in an emergency”, α = 0.89) and Negative (four items, “There is not an adult I can turn to for guidance in times of stress”, α = 0.76) (Phillips & Springer, 1992).

**School Domain.** School safety problems were assessed by two scales (response options: 1 = *Not a problem* to 4 = *Serious problem*): Violence and Firearms (seven items, e.g., “Students carrying guns”, α *=* 0.92) and Teacher Behavior (three items, e.g., “Teachers not knowing what students are up to”, α *=* 0.77; Multisite Violence Prevention Project, 2004). School climate (six items, e.g., “Teachers take a personal interest in students”, α = 0.71) was measured on a scale of 1 (*Strongly disagree*) to 5 (*Strongly agree*) (Brand et al., 2003).

**Family Domain.** The Expanded Family ACEs scale assessed childhood exposure to family adversity (nine items, e.g., “Did your parents or guardians ever get separated or divorced?”; National Survey of Children’s Health, 2017). Participants responded *yes* or *no* to three questions, generated through participatory action research, that assessed whether any close family members had committed a violent crime or been injured or killed due to violence (Mehari et al., 2026a). Family conflict was measured by an average rating of six items (e.g., “My parent screams a lot”, α = 0.82; 1 = *Strongly disagree* to 5 = *Strongly agree*) (Prinz et al., 1979). Household firearm access was assessed by one item, “Do you currently have a firearm or firearms?” (*No* [no access]; *Yes* [personal ownership]; *There’s a firearm where I live, but it’s not mine* [household ownership]). Living with their biological father was assessed by a checklist identifying everyone who lived in the home.

**Peer Domain.** Participants responded *Yes* or *No* to three questions generated through participatory action research that assessed whether any friends had committed a violent crime or been injured or killed due to violence (Mehari et al., 2026b). Friend firearm carrying was assessed by one item asking how many of their friends carried firearms (1 = *None of them* to 4 = *All of them*; Conduct Problems Prevention Research Group, 2010). Using the same scale, friends’ delinquent behavior was assessed by asking how many of the participants’ close friends engaged in six behaviors (e.g., “Sold drugs;” α = 0.79) (Farrell et al., 2017).

**Intra-Individual Domain.** Youths’ problem behaviors were assessed by past 30-day substance use (seven items, e.g., “Been drunk”), physical aggression (five items, e.g., “Shoved or pushed someone”), and physical victimization (four items; e.g., “Someone pushed or shoved you”) from the Problem Behavior Frequency Scales (1 = *Never* to 4 = *6–9 times*, αs ranged from 0.80 to 0.88; Farrell et al., 2016). Lifetime firearm-related victimization was assessed by three items (ever having been threatened, shot at, or injured with a firearm) and was coded to reflect the presence or absence of firearm-related victimization (Bureau of Justice Statistics, n.d.; Martinez & Richters, 1993; Richters & Saltzman, 1990). Youths’ mental health indicators included traumatic stress symptoms (12 items, 1 = *Never* to 5 = *Most of the time*, e.g., “Do you worry about being safe?”, α = 0.90), anger dysregulation (five items, 1 = *Hardly Ever* to 3 = *Often*, e.g., “When I get angry, I explode”, α = 0.76), and positive outlook (six items; 1 = *NO!* to 4 = *YES!*, e.g., “I think I will have a nice family when I get older”, α = 0.73) (Phillips & Springer, 1992; Richters & Martinez, 1990; Zeman et al., 2001).

Measures of youths’ firearm-related attitudes and beliefs were developed through participatory action research (Mehari et al., 2026a, 2025b). General Attitudes Toward Firearms included Positive Attitudes (five items, e.g., “Guns are necessary”, α = 0.83) and Negative Attitudes (three items, e.g., “Guns are scary”, α = 0.74). Beliefs About Defensive Firearm Use included three subscales (response options: 1 = *Strongly disagree* to 5 = *Strongly agree*): Respect Enforcement (four items, e.g., “If someone killed a person who you’re close to, it’s okay to go kill them”, α = 0.87), Compensatory Force (three items, e.g., “If someone makes you feel threatened, it’s okay to tell them you have a gun”, α = 0.61), and Defensive Escalation (two items, e.g., “If you see someone getting attacked, it’s okay to shoot the attacker”, α = 0.80). Reasons for storage was a checklist with two subscales: Self-defense (three items, e.g., “To protect myself”, α = 0.89) and Inherent danger (four items, e.g., “Keep accidents from happening”, α = 0.84).

**Firearm-Related Violence Perpetration.** The three items used to assess youths’ firearm use against others (ever shot at someone, ever used a gun against someone, or used a gun to defend yourself) were coded into a dichotomous variable, with 1 indicating any violent firearm use and 0 indicating no violent firearm use.

**Demographics.** Participants provided their age, race or ethnicity, sex assigned at birth, sexual orientation, and gender identity (LGBTQIA+ Health Education Center, n.d.). Sexual or gender minority status was classified as present if a youth identified with a gender that differed from their sex assigned at birth or indicated a sexual orientation outside of “straight or heterosexual,” excluding any open-ended responses consistent with trolling (e.g., “civil attack helicopter”). School-level poverty was a binary variable, with high-poverty schools defined as those where over 75% of students qualified for FRPL (National Center for Education Statistics, n.d.). Three dummy variables were created to represent the school at which participants were enrolled, with the fourth serving as the reference school.

### 2.5. Analysis Plan

Mplus version 8.10 (Muthén & Muthén, 2017) was used for all analyses. Variables with a skewed and kurtotic distribution (i.e., substance use, physical aggression, physical victimization, exposure to community violence, friends’ delinquent behavior) were log-transformed. Data that were missing at random were addressed using full information maximum likelihood estimation with robust standard errors (mlr in Mplus). Across all youth-reported variables, missing data ranged from 1% to 7%, with the exception of firearm-related violence victimization, at 18%. Missing data for firearm-related victimization occurred because the item that assessed whether youth had been threatened with a firearm was not included in the pilot school survey, so this variable was not calculated for any participant attending the pilot school. We tested the final model for the full sample and for the pilot sample separately to examine whether this difference impacted the results (see Supplementary Tables S2 and S3). Except for age and number of ACEs, continuous variables were standardized to facilitate interpretation in all regression analyses. A series of bivariate logistic regression models tested the relation between each independent variable and violent firearm use. Variables that were not associated with violent firearm use were excluded from subsequent analyses. Multivariate logistic regression models examined the unique (partial regression coefficient) and combined (*R*^2^) effects of independent variables within each social-ecological level on violent firearm use. For these and subsequent analyses, intra-individual level variables were grouped into three sets including problem behavior, mental health, and beliefs and attitudes, which enabled a more nuanced examination of their unique and combined effects on violent firearm use. In the final model, all independent variables were entered simultaneously. A Wald test was used to determine the significance of the combined effects of the variables within each social-ecological level on violent firearm use.

## 3. Results

Correlations among the variables are available in Supplementary Table S1. Violent use of firearms was reported by 11.2% of the sample (95% confidence interval: 9.3–13.1%). Nearly all the hypothesized predictors exhibited significant bivariate associations with violent firearm use in the expected direction (see Table 1). The variables that were not associated with violent firearm use were sexual/gender minority status, household firearm ownership, positive outlook, beliefs supporting the use of firearms for defensive escalation, negative attitudes about firearms, and positive attitudes about firearms. Consistent with the model-building approach, these variables were excluded from subsequent analyses. However, we conducted a supplemental full model including all hypothesized predictors to test the robustness of our findings and to explore whether including the nonsignificant variables changed the patterns of results. Results of this model revealed patterns that were largely the same as in the model which included only the predictors that emerged as statistically significant. This model is available in Supplementary Materials, Table S2.

### 3.1. Predicting Violent Firearm Use Within Each Social-Ecological Domain

The results of multivariate models testing the extent to which each social-ecological level explained youths’ violent firearm use are reported in Table 2. Among demographic variables, male youth had twice the odds of engagement compared to female youth, and youth in high-poverty schools had over triple the odds of engagement. Among cultural factors, media exposure to violence and guns was associated with 47% higher odds of violent firearm use. Among community-level factors, greater exposure to community violence and the absence of a caring adult were associated with 77% and 39% higher odds, respectively, whereas higher neighborhood collective efficacy and caring adult support were associated with decreased odds. Among school-level factors, perceived violence at school was associated with 49% increased odds, whereas positive school climate was associated with 24% decreased odds. Among family-level factors, higher ACEs and having a family member injured or killed by violence were associated with greater odds of violent firearm use (12% and 62%, respectively). Among peer-level variables, having a friend who committed (179%) or experienced (92%) violent injury was associated with increased risk, as did friends’ delinquent behavior (23%). Among intra-individual problem behavior, mental health, and firearm beliefs, substance use was associated with 54% higher odds, firearm-related victimization with 473% higher odds, traumatic stress with 28% higher odds, and beliefs supporting firearm use for respect enforcement with 65% higher odds. Beliefs in safe storage to prevent harm was associated with 42% decreased odds.

### 3.2. Full Model Predicting Violent Firearm Use Across Social-Ecological Domains

The full model tested all social-ecological variables simultaneously (*R*^2^ = 0.41, *p* < 0.001; see Table 3 for results). Variables at the demographic and intra-individual (i.e., problem behavior, firearm beliefs) levels combined explained a significant proportion of the variance in violent firearm use. Male sex was the only demographic factor uniquely associated, with a 135% increase in odds compared to female sex. Having a friend who committed a violent crime was associated with about double the odds of violent use of firearms. Substance use was associated with 63% higher odds. Beliefs supporting compensatory firearm use was associated with 44% higher odds, whereas beliefs prioritizing storage that protected against the inherent danger of firearms was associated with lower odds of violent firearm use. Notably, firearm-related victimization was not significantly associated with violent firearm use when controlling for school. Other domains did not have significant unique relations with youths’ violent firearm use. The full model was also tested for the pilot sample separately due to differences in how firearm-related victimization was assessed; this model can be found in the Supplementary Materials, Table S3.

## 4. Discussion

The purpose of this study was to examine the extent to which factors across intra-individual, peer, family, school, neighborhood, and cultural domains were associated with youths’ violent firearm use. Of the 37 hypothesized factors, 30 had significant bivariate associations with violent firearm use. Altogether, the hypothesized social-ecological factors explained 42% of the variance in youths’ violent firearm use. Factors in the demographic, intra-individual, and peer domains were uniquely associated with violent firearm use in the full model. Overall, these findings provide novel insights about youths’ violent use of firearms. The findings validate the relevance of factors identified by community members prior to data collection, highlighting the critical role of community-informed hypothesis testing in understanding and addressing youth violent firearm use.

Male sex, firearm-related victimization, substance use, firearm-related beliefs, and having a friend who committed a violent crime were uniquely associated with youths’ violent firearm use after accounting for the other social-ecological variables. Previous research has also identified male youth as having a higher risk for involvement in violent firearm use compared to female youth (Pulavarthi et al., 2024; Stoddard et al., 2011), which may be related to both biological factors and gender socialization, including male youths’ greater exposure to frequent and severe violence experiences compared to their female counterparts (Stoddard et al., 2011). The consistent nature of this finding suggests that gender differences in violence trajectories emerge early and endure across development (Tremblay et al., 2004).

One pattern that emerged across findings was that the violent use of firearms likely occurs in the context of violence exposure across domains (Goldstick et al., 2019; McGee et al., 2017). Specifically, firearm-related victimization was associated with 175% higher odds of violent firearm use in the model testing individual-level problem behavior. Additionally, having a friend who engaged in a violent crime explained unique variance in the full model, associated with double the odds of violent firearm use. When not controlling for school, firearm-related victimization was associated with significantly increased odds of violent firearm use. When including school in the full model, firearm-related victimization did not explain unique variance; however, it may still be associated with youths’ violent firearm use. This finding highlights in the important role that contextual factors specific to youths’ school environment (e.g., enrollment at a historically segregated and/or high-poverty school) play in the relation between violence exposure and violent firearm use. Similarly, although they did not explain unique variance when controlling for all other factors, being exposed to community violence, perceiving violence at school, and having a family member or friend injured or killed by violence were all associated with greater odds of violent firearm use. These findings align with research showing that repeated exposure to violence in community, school, peer, and home environments are associated with increased likelihood of firearm carrying and violent firearm use (Schmidt et al., 2019). Exposure to violence can erode youths’ sense of safety and destabilize social support, and it may be linked to limited social and economic resources, particularly within their community and social networks.

One potential promotive factor that was associated with lower odds of engaging in violent firearm use was the belief that firearm storage practices should prioritize reducing the risk associated with having a firearm in the home. Youth who prioritize safety-related decision-making are less likely to engage in violent firearm use, suggesting that youth who respect the inherent danger of firearms may be more likely to avoid situations that may place them at risk for violence involvement. It is also possible that these are messages that youth receive from their parents, reflecting a broader family culture of safety. Of note, although we expected that the presence of a caring adult would be a promotive factor, it was the *absence* of a caring adult that was associated with violent firearm use. Importantly, youth who do *not* feel supported and cared for by at least one reliable adult may be more likely to engage in violence, supporting the importance of family-based, mentorship-based, and school climate-focused interventions so that fewer youth experience the absence of a caring adult.

Social-ecological factors at the cultural, community, school, and family levels did not predict unique variance in the final model. However, these variables did show bivariate associations with violent use of firearms, and many of them explained unique variance in the domain-specific models (e.g., media exposure to violence and firearms; community violence exposure; neighborhood safety; caring adult presence; school safety problems; school climate; ACEs; death or injury of a family member due to violence), suggesting that these are still important factors related to violent use of firearms. It is possible that more proximal factors such as peer group behaviors and individual-level beliefs and behaviors fully mediate the more distal contextual influences, but this is a hypothesis that was not tested in the current study. Future longitudinal research should explore the extent to which proximal factors explain the relations between contextual violence and youths’ violent use of firearms. Additionally, the limited number of schools made it impossible for us to control for clustering at the school level, which would have been a stronger approach to examine contextual effects. For example, being in a high-poverty school no longer explained unique variance in the full model, perhaps because it explained overlapping variance with other factors at the school- and community level. School poverty levels are linked to racial segregation, a deliberate and systemic government practice that resulted in concentrated poverty and divestment of resources (Rothstein, 2017). Such predictors include racial segregation and income segregation which simultaneously protect White Americans from firearm violence and disproportionately place Black Americans at risk (Goin et al., 2018; Kim, 2019; Light & Thomas, 2019). We were unable to assess these and race- and place-based inequities that are predictive of firearm violence, such as environmental hazards, physical disorder, and open-air drug markets (Bingenheimer et al., 2005; Howard et al., 2000; Kennedy et al., 1996).

Notably, at the intra-individual level, mental health factors as a group did not explain a significant amount of the variance in violent firearm use, although posttraumatic stress was individually related to violent firearm use. There is some evidence to suggest that posttraumatic stress disorder and substance use disorders are risk factors for firearm-related victimization and violent firearm use (Carter et al., 2017; Goldstick et al., 2019). However, the current findings indicate that youths’ violent firearm use may be better explained by other social-ecological factors. Future research should examine posttraumatic stress as a potential mediator of the relation between violent exposure and victimization and youths’ violent firearm use.

### 4.1. Limitations and Future Directions

The cross-sectional design of this study limits the possibility of identifying the temporal order of associations; that is, we cannot know whether our hypothesized predictors came before or after youths’ violent use of firearms. We cannot therefore draw any conclusion about what preceded or caused what, and it is possible that what we hypothesized to be predictors were actually caused *by* youths’ violent firearm use, or that unmeasured factors caused both our social-ecological factors and youths’ violent firearm use. The generalizability of the results is limited given that all youth participants were recruited from one county in the Southeastern U.S. It is likely that patterns of violent firearm use are impacted by context; for example, the relation between firearm-related victimization and violent firearm use may be stronger in under-resourced neighborhoods (Berg & Loeber, 2011). The study data are representative of highly segregated, highly burdened, divested communities in the Southeastern U.S., which is characteristic of predominantly Black Southern communities. As such, these data are likely representative of other segregated communities that are highly burdened by firearm-related violence. The violent use of firearms index used in this study was developed through a participatory research process (Mehari et al., 2026a), and it combines both self-defense and proactive use to create a dichotomous indicator. These behaviors may be distinct and have unique predictors that could not be explored in this study. Another limitation is that all social-ecological domain variables were assessed through self-report, essentially assessing youths’ perceptions of their contexts rather than providing objective indicators. However, youths’ perceptions provide valuable information and do serve as indicators of their context. For example, youths’ reports of friends’ delinquent behaviors are predictive of their friends’ self-reported delinquent behaviors (Boman et al., 2012).

Other possible limitations include selection bias; for example, school administrators may have made decisions about participating due to the relevance of the topic area. Youth with organizational strengths (e.g., bringing the consent form to parents or guardians and bringing it back to the school) may also have been more likely to participate. However, controlling for the school in the final model may have mitigated bias related to the specific schools that participated. Another potential limitation is that the estimates of the correlations among variables may have been inflated due to monomethod bias (i.e., all variables were self-reported).

Future research would benefit from integrating longitudinal and multimethod approaches to provide a more robust understanding of the relations suggested by these results. In addition, future research should examine the potential complex interactions across social-ecological domains that may be important in understanding the violent use of firearms (e.g., factors that may exacerbate or mitigate risk; potential mechanisms through which these risk factors exert their effects).

### 4.2. Conclusions

This study represents an important step in expanding the existing understanding of possible predictors of youths’ violent use of firearms. Though longitudinal work is needed, these emerging findings support research that suggests that multisystemic interventions, rather than interventions focused on youth alone, may provide the strongest benefits (Thompson et al., 2026). Specifically, exposure to violence in the community, in schools, in friend groups, and in families may be particularly important risks and therefore particularly important targets of intervention. Interventions intended to reduce firearm violence should include reducing youths’ exposure to violence across domains and mitigate the potential ripple effects of that exposure (Mehari et al., 2025a). Those interventions can include structural interventions, such as vacant lot remediation and greening (Sadatsafavi et al., 2022), school climate interventions to improve relationships within schools and promote safety (Catalano et al., 1999; Sullivan et al., 2025), mentoring interventions to increase access to caring adults (e.g., Jones et al., 2026), interventions that increase opportunity, such as youth employment programs (Joseph & Jay, 2026), and intervention to increase prosocial support in families and friend groups. This study also points to the importance of addressing individual behaviors and beliefs in preventing youth firearm-related violence, particularly through intervention strategies that enhance youths’ strengths and resources, including beliefs about firearms (e.g., Edberg et al., 2026), to mitigate factors that may increase their risk of firearm-related violence involvement, such as substance use.

## Figures and Tables

**Table 1:** Descriptive Statistics and Bivariate Associations Between Each Independent Variable and Youths’ Violent Firearm Use.

Measure	*n* Endorsed (% Endorsed)	Mean ^a^ (SD)	*b*	*OR* (95% CI)
Demographics				
Male sex	586 (54.77)		0.69 **	1.98 (1.34–2.94)
Age		16.17 (1.15)	0.35 ***	1.42 (1.22–1.66)
High-poverty school	616 (60.63)		1.45 ***	4.25 (2.73–6.6)
White race ^b^	354 (33.59)		−1.34 ***	0.26 (0.15–0.47)
Other race ^b^	164 (15.56)		−0.24	0.79 (0.46–1.35)
Sexual and/or gender minority	180 (18.44)		−0.01	0.99 (0.58–1.7)
Cultural level				
Media exposure to violence and guns ^c^		3.41 (0.93)	0.39 ***	1.47 (1.19–1.83)
Community level				
Exposure to community violence via witnessing ^c^		1.75 (0.95)	0.60 ***	1.83 (1.55–2.16)
Neighborhood safety and cohesion ^c^		3.29 (0.70)	−0.37 ***	0.69 (0.58–0.83)
Presence of a caring adult—Positive items ^c^		3.26 (0.72)	−0.34 ***	0.71 (0.6–0.85)
Presence of a caring adult—Negative items ^c^		2.02 (0.75)	0.40 ***	1.50 (1.24–1.81)
School level				
School Safety Problems—Violence and Guns ^c^		2.14 (0.98)	0.35 ***	1.42 (1.21–1.67)
School Safety Problems—Teacher Behavior ^c^		2.35 (0.89)	0.24 **	1.27 (1.07–1.51)
School Climate ^c^		3.55 (0.62)	−0.15 **	0.02 (1.41–0.16)
Family level				
Number of adverse childhood experiences		2.53 (2.17)	0.19 ***	1.21 (1.12–1.31)
Close family member committed a violent crime	278 (24.76)		0.84 ***	2.31 (1.56–3.42)
Close family member injured or killed due to violence	545 (48.49)		0.69 **	2 (1.35–2.96)
Family conflict ^c^		2.70 (0.88)	0.19 *	1.21 (1.03–1.44)
Household gun possession	740 (68.39)		0.13	1.14 (0.75–1.73)
Lives with biological father	483 (42.90)		−0.57 **	0.57 (0.38–0.84)
Peer level				
Friend committed a violent crime	121 (10.77)		1.69 ***	5.42 (3.48–8.44)
Friend injured or killed due to violence	426 (37.93)		1.10 ***	3.00 (2.04–4.40)
Friend carried a gun	188 (16.73)		1.19 ***	3.28 (2.18–4.94)
Friends’ delinquent behavior ^c^		1.26 (0.43)	0.48 ***	1.62 (1.38–1.90)
Intra-individual level–Problem behavior				
Substance use ^c^		1.44 (0.70)	0.62 ***	1.86 (1.58–2.20)
Physical aggression ^c^		1.73 (0.81)	0.54 ***	1.72 (1.40–2.10)
Physical victimization ^c^		1.51 (0.70)	0.40 ***	1.49 (1.24–1.78)
Firearm victimization	219 (23.70)		2.13 ***	8.37 (5.32–13.18)
Intra-individual level–Mental health				
Traumatic stress ^c^		2.56 (0.93)	0.28 **	1.32 (1.11–1.58)
Dysregulated anger expression ^c^		1.67 (0.49)	0.18 *	1.19 (1.00–1.42)
Positive outlook ^c^		3.14 (0.58)	−0.13	0.88 (0.73–1.05)
Intra-individual level–Beliefs and attitudes				
Beliefs about Firearms—Defensive escalation ^c^		3.10 (1.03)	0.07	1.07 (0.88–1.32)
Beliefs about Firearms—Compensatory force ^c^		2.67 (0.88)	0.32 **	1.38 (1.12–1.69)
Beliefs about Firearms—Respect enforcement ^c^		2.09 (0.86)	0.64 ***	1.89 (1.55–2.29)
Reasons for storage—Self-defense ^c^		3.70 (1.07)	−0.30 **	−0.16 (−3.51–0.00)
Reasons for storage—Inherent danger ^c^		3.89 (1.02)	−0.47 ***	0.63 (0.54–0.74)
Negative Attitudes Toward Firearms ^c^		2.97 (1.01)	−0.11	0.90 (0.75–1.08)
Positive Attitudes Toward Firearms ^c^		3.17 (0.99)	−0.04	0.96 (0.78–1.17)

*Note.* SD = Standard Deviation; OR = Odds Ratio. ^a^ Unstandardized mean. ^b^ Reference group = Black race. ^c^ Variable was standardized in bivariate regression analysis to facilitate interpretation. * *p* < 0.05. ** *p* < 0.01. *** *p* < 0.001.

**Table 2:** Multivariate Associations Between Variables at Each Social-Ecological Level and Youths’ Violent Firearm Use.

Measure	*R* ^2^	*b*	*AOR* (95% CI)
Demographics	0.20 ***		
Male sex		0.70 **	2.01 (1.34–3.02)
Age		0.27 **	1.31 (1.11–1.54)
High-poverty school		1.23 ***	3.43 (1.80–6.52)
White race ^a^		−0.34	0.71 (0.31–1.64)
Other race ^a^		0.18	1.20 (0.65–2.21)
Cultural level	0.04		
Media exposure to violence and guns ^b^		0.39 ***	1.47 (1.19–1.83)
Community level	0.17 ***		
Exposure to community violence via witnessing ^b^		0.57 ***	1.77 (1.49–2.1)
Neighborhood safety and cohesion ^b^		−0.22 *	0.80 (0.65–0.99)
Presence of a caring adult–Positive items ^b^		−0.16	0.86 (0.69–1.06)
Presence of a caring adult–Negative items ^b^		0.33 **	1.39 (1.11–1.75)
School level	0.06 *		
School Safety Problems–Violence and Guns ^b^		0.40 ***	1.49 (1.19–1.86)
School Safety Problems–Teacher Behavior ^b^		−0.08	0.93 (0.73–1.18)
School Climate ^b^		−0.27 **	0.76 (0.63–0.93)
Family level	0.08 **		
Number of adverse childhood experiences		0.11 *	1.12 (1.01–1.25)
Close family member committed a violent crime		0.23	1.26 (0.81–1.94)
Close family member injured or killed due to violence		0.48 *	1.62 (1.04–2.53)
Family conflict ^b^		0.03	1.03 (0.85–1.25)
Lives with biological father		−0.31	0.74 (0.48–1.13)
Peer level	0.13 ***		
Friend committed a violent crime		1.03 ***	2.79 (1.70–4.59)
Friend injured or killed due to violence		0.65 **	1.92 (1.26–2.92)
Friend carried a gun		0.35	1.42 (0.85–2.34)
Friends’ delinquent behavior ^b^		0.21 *	1.23 (1.02–1.49)
Intra-individual level–Problem behavior	0.21 ***		
Substance use ^b^		0.43 ***	1.54 (1.28–1.86)
Physical aggression ^b^		0.06	1.06 (0.82–1.37)
Physical victimization ^b^		−0.16	0.85 (0.68–1.06)
Firearm-related victimization		1.75 ***	5.73 (3.55–9.26)
Intra-individual level–Mental health	0.03		
Traumatic stress ^b^		0.24 **	1.28 (1.06–1.53)
Dysregulated anger expression ^b^		0.12	1.13 (0.93–1.36)
Intra-individual level–Beliefs and attitudes	0.18 ***		
Beliefs about Firearms–Compensatory force ^b^		0.21	1.24 (0.89–1.71)
Beliefs about Firearms–Respect enforcement ^b^		0.50 ***	1.65 (1.28–2.11)
Reasons for storage–Self-defense ^b^		0.02	1.02 (0.73–1.43)
Reasons for storage–Inherent danger ^b^		−0.54 **	0.58 (0.43–0.79)

*Note. R*^2^ is provided by domain, and *b* is provided for single factors. *R*^2^ is not a traditional pseudo- *R*^2^ statistic and is produced in Mplus by estimating the variance explained in the latent response variable that underlies the categorical variable. AOR = Adjusted Odds Ratio; CI = Confidence Interval. ^a^ Reference group = Black race. ^b^ Variable was standardized to facilitate interpretation. * *p* < 0.05. ** *p* < 0.01. *** *p* < 0.001.

**Table 3:** Full Multivariate Model of Social-Ecological Predictors of Youths’ Violent Firearm Use.

Measure	χ^2^	*b*	*AOR* (95% CI)
Demographics	29.27 ***		
Male sex		0.85 ***	2.35 (1.46–3.77)
Age		0.20	1.22 (0.98–1.51)
Enrollment at School A ^a^		0.99	2.69 (0.97–7.47)
Enrollment at School B ^a^		0.62	1.86 (0.75–4.63)
Enrollment at School C ^a^		−0.38	0.68 (0.27–1.75)
White race ^b^		−0.12	0.89 (0.35–2.22)
Other race ^b^		−0.18	0.83 (0.41–1.71)
Cultural level	2.68		
Media exposure to violence and guns ^c^		0.21	1.24 (0.96–1.59)
Community level	8.44		
Exposure to community violence via witnessing ^c^		0.22	1.25 (0.99–1.59)
Neighborhood safety and cohesion ^c^		−0.18	0.84 (0.67–1.06)
Presence of a caring adult–Positive items ^c^		0.05	1.05 (0.82–1.35)
Presence of a caring adult–Negative items ^c^		0.21	1.23 (0.96–1.57)
School level	0.86		
School Safety Problems–Violence and Guns ^c^		−0.05	0.95 (0.68–1.32)
School Safety Problems–Teacher Behavior ^c^		0.10	1.07 (0.93–1.22)
School Climate ^c^		−0.05	0.95 (0.75–1.25)
Family level	1.80		
Number of adverse childhood experiences		0.06	1.06 (0.92–1.21)
Close family member committed a violent crime		−0.22	0.81 (0.48–1.36)
Close family member injured or killed due to violence		−0.11	0.89 (0.52–1.54)
Family conflict ^c^		−0.05	0.95 (0.73–1.25)
Lives with biological father		0.19	1.22 (0.73–2.02)
Peer level	7.66		
Friend committed a violent crime		0.69 *	1.99 (1.13–3.53)
Friend injured or killed due to violence		0.29	1.34 (0.83–2.17)
Friend carried a gun		−0.08	0.92 (0.52–1.64)
Friends’ delinquent behavior ^c^		0.01	1.01 (0.79–1.29)
Intra-individual level–Problem behavior	17.25 **		
Substance use ^c^		0.49 ***	1.63 (1.24–2.14)
Physical aggression ^c^		−0.07	0.94 (0.70–1.29)
Physical victimization ^c^		−0.09	0.81 (0.63–1.05)
Firearm-related victimization		0.61	1.84 (0.77–4.42)
Intra-individual level–Mental health	1.35		
Traumatic stress ^c^		0.09	1.10 (0.83–1.46)
Dysregulated anger expression ^c^		−0.13	0.88 (0.68–1.14)
Intra-individual level–Beliefs and attitudes	18.87 **		
Beliefs about Firearms–Compensatory force ^c^		0.37 *	1.44 (1.02–2.03)
Beliefs about Firearms–Respect enforcement ^c^		0.14	1.15 (0.87–1.52)
Reasons for storage–Self-defense ^c^		−0.13	0.88 (0.61–1.27)
Reasons for storage–Inherent danger ^c^		−0.39 *	0.68 (0.49–0.95)

*Note.* χ^2^ is provided by domain, and *b* is provided for single factors. *R*^2^ is not a traditional pseudo- *R*^2^ statistic and is produced in Mplus by estimating the variance explained in the latent response variable that underlies the categorical variable. AOR = Adjusted Odds Ratio; CI = Confidence Interval. ^a^ Reference group = School D. ^b^ Reference group = Black race. ^c^ Variable was standardized to facilitate interpretation. * *p* < 0.05. ** *p* < 0.01. *** *p* < 0.001.

## Data Availability

All data are available upon reasonable request from the corresponding author following a Data Use Agreement and IRB approval.

## Note

1 Throughout this manuscript, we use “violent firearm use” instead of “perpetration” for multiple reasons. First, “perpetration” is associated with stigma, especially against Black boys and young men, which we do not wish to perpetuate. Second, “perpetration” often has criminal and legal connotations, which is not the purpose of this study. Finally, per our advisory board discussions, many individuals who use firearms against others believe that they are acting in their own defense or in the defense of others, which is not consistent with “perpetration” language.

## Supplementary Materials

The following supporting information can be downloaded at: https://www.mdpi.com/article/10.3390/bs16101916/s1, Table S1: Correlations Among Variables; Table S2: Full Multivariate Model with All Hypothesized Social-Ecological Predictors of Youths’ Violent Firearm Use; Table S3: Full Multivariate Model of Social-Ecological Predictors of Youths’ Violent Firearm Use Among the Pilot Sample Only.

## References

[B1-behavsci-16-01916] Bender A. K., Koegler E., Johnson S. D., Murugan V., Wamser-Nanney R. (2021). Guns and intimate partner violence among adolescents: A scoping review. Journal of Family Violence.

[B2-behavsci-16-01916] Berg M. T., Loeber R. (2011). Examining the neighborhood context of the violent offending-victimization relationship: A prospective investigation. Journal of Quantitative Criminology.

[B3-behavsci-16-01916] Bingenheimer J. B., Brennan R. T., Earls F. J. (2005). Firearm violence exposure and serious violent behavior. Science.

[B4-behavsci-16-01916] Boman J. H., Stogner J. M., Miller B. L., GriffinIII O. H., Krohn M. D. (2012). On the operational validity of perceptual peer delinquency: Exploring projection and elements contained in perceptions. Journal of Research in Crime and Delinquency.

[B5-behavsci-16-01916] Brand S., Felner R., Shim M., Seitsinger A., Dumas T. (2003). Middle school improvement and reform: Development and validation of a school-level assessment of climate, cultural pluralism, and school safety. Journal of Educational Psychology.

[B6-behavsci-16-01916] Bronfenbrenner U. (1977). Toward an experimental ecology of human development. American Psychologist.

[B7-behavsci-16-01916] Bureau of Justice Statistics (n.d.). National crime victimization survey (NCVS).

[B8-behavsci-16-01916] Carter P. M., Walton M. A., Goldstick J., Epstein-Ngo Q. M., Zimmerman M. A., Mercado M. C., Williams A. G., Cunningham R. M. (2017). Violent firearm-related conflicts among high-risk youth: An event-level and daily calendar analysis. Preventive Medicine.

[B9-behavsci-16-01916] Catalano R. F., Loeber R., McKinney K. C. (1999). School and community interventions to prevent serious and violent offending *(No. 177624; OJJDP Serious and Violent Juvenile Offending Series)*.

[B10-behavsci-16-01916] Centers for Disease Control and Prevention (n.d.). Underlying cause of death, 2018–2022, single race results.

[B11-behavsci-16-01916] Centers for Disease Control and Prevention (2021). Fatal injury data: Leading causes of death.

[B12-behavsci-16-01916] Conduct Problems Prevention Research Group (2010). The effects of a multiyear universal social–emotional learning program: The role of student and school characteristics. Journal of Consulting and Clinical Psychology.

[B13-behavsci-16-01916] Edberg M. C., Evans W. D., Wang Y., Andrade E. L., Sachdev N., Long M. W., Manso L., Sciortino M., Wallace M., Tutt J., Battle V., Rioland K., Bryant D., Mack A. (2026). The run it up intervention: Addressing the effects of structural determinants on adolescent identity, beliefs, and involvement in firearm violence—Formative research and intervention development. Prevention Science.

[B14-behavsci-16-01916] Erickson P. G., Butters J. E., Cousineau M.-M., Harrison L., Korf D., Drugs, Alcohol and Violence International (DAVI) Team (2006). Girls and weapons: An international study of the perpetration of violence. Journal of Urban Health.

[B15-behavsci-16-01916] Farrell A. D., Sullivan T. N., Goncy E. A., Le A.-T. H. (2016). Assessment of adolescents’ victimization, aggression, and problem behaviors: Evaluation of the problem behavior frequency scale. Psychological Assessment.

[B16-behavsci-16-01916] Farrell A. D., Thompson E. L., Mehari K. R. (2017). Dimensions of peer influences and their relationship to adolescents’ aggression, other problem behaviors and prosocial behavior. Journal of Youth and Adolescence.

[B17-behavsci-16-01916] Goin D. E., Rudolph K. E., Ahern J. (2018). Predictors of firearm violence in urban communities: A machine-learning approach. Health & Place.

[B18-behavsci-16-01916] Goldstick J. E., Carter P. M., Heinze J. E., Walton M. A., Zimmerman M., Cunningham R. M. (2019). Predictors of transitions in firearm assault behavior among drug-using youth presenting to an urban emergency department. Journal of Behavioral Medicine.

[B19-behavsci-16-01916] Green B., Horel T., Papachristos A. V. (2017). Modeling contagion through social networks to explain and predict gunshot violence in Chicago, 2006 to 2014. JAMA Internal Medicine.

[B20-behavsci-16-01916] Howard G., Anderson R. T., Russell G., Howard V. J., Burke G. L. (2000). Race, socioeconomic status, and cause-specific mortality. Annals of Epidemiology.

[B21-behavsci-16-01916] Huesmann L. R., Dubow E. F., B. Boxer P., Bushman B. J., S. Smith C., A. Docherty M., J. O’Brien M. (2021). Longitudinal predictions of young adults’ weapons use and criminal behavior from their childhood exposure to violence. Aggressive Behavior.

[B22-behavsci-16-01916] Intravia J., Wolff K. T., Paez R., Gibbs B. R. (2017). Investigating the relationship between social media consumption and fear of crime: A partial analysis of mostly young adults. Computers in Human Behavior.

[B23-behavsci-16-01916] Jamieson P. E., Romer D. (2021). The association between the rise of gun violence in popular US primetime television dramas and homicides attributable to firearms, 2000–2018. PLoS ONE.

[B24-behavsci-16-01916] Jones K., Duckworth J., Fetters C., Galoro B., Rowhani-Rahbar A. (2026). Been there, done that, and now I’m giving back: Perspectives from mentors and administrators in community violence intervention programs. Prevention Science.

[B25-behavsci-16-01916] Joseph P. L., Jay J. (2026). Summer youth employment programs as a structural approach to prevent youth violence: An Integrative review. Prevention Science.

[B26-behavsci-16-01916] Kennedy D. M., Piehl A. M., Braga A. A. (1996). Youth violence in Boston: Gun markets, serious youth offenders, and a use-reduction strategy. Law and Contemporary Problems.

[B27-behavsci-16-01916] Kim D. (2019). Social determinants of health in relation to firearm-related homicides in the United States: A nationwide multilevel cross-sectional study. PLoS Medicine.

[B28-behavsci-16-01916] Lee D. B., Schmidt C. J., Heinze J. E., Carter P. M., Cunningham R. M., Walton M. A., Zimmerman M. A. (2022). Retaliatory attitudes as mediator of exposure to violence and firearm aggression among youth: The protective role of organized activity involvement. Developmental Psychology.

[B29-behavsci-16-01916] LGBTQIA+ Health Education Center (n.d.). Ready, set, go! Guidelines and tips for collecting patient data on sexual orientation and gender identity (SOGI)—2022 update.

[B30-behavsci-16-01916] Light M. T., Thomas J. T. (2019). Segregation and violence reconsidered: Do whites benefit from residential segregation?. American Sociological Review.

[B31-behavsci-16-01916] Loeffler C., Flaxman S. (2018). Is gun violence contagious? A spatiotemporal test. Journal of Quantitative Criminology.

[B32-behavsci-16-01916] Martinez P., Richters J. E. (1993). The nimh community violence project: II. Children’s distress symptoms associated with violence exposure. Psychiatry.

[B33-behavsci-16-01916] McGee Z. T., Logan K., Samuel J., Nunn T. (2017). A multivariate analysis of gun violence among urban youth: The impact of direct victimization, indirect victimization, and victimization among peers. Cogent Social Sciences.

[B34-behavsci-16-01916] Mehari K. R., Farrell A. D., Coleman J. N., Mayinja L. S., Walsh C. S., Smith P. N. (2026a). Community-based development of tools to assess firearm-related attitudes and beliefs: Associations with firearm-related practices, social influences, and violence exposure. Journal of Community Health.

[B35-behavsci-16-01916] Mehari K. R., Smith P. N., Dunn C., Coleman J. N., Stevens L. T. (2025a). Prevalence and patterns of nonfatal firearm-related violence among adolescents in the southeastern United States. Psychology of Violence.

[B36-behavsci-16-01916] Mehari K. R., Smith P. N., Farrell A. D. (2025b). Project GRIP youth survey codebook *[Unpublished]*.

[B37-behavsci-16-01916] Mehari K. R., Walsh C. S., Taylor Stevens L., Coleman J. N., Ellyson A., Smith P. N. (2026b). Firearm carrying practices and motivations: A mixed methods study among adults and youth. American Journal of Community Psychology.

[B38-behavsci-16-01916] Multisite Violence Prevention Project (2004). Description of measures: Cohort-wide student survey *(unpublished)*.

[B39-behavsci-16-01916] Muthén L. K., Muthén B. O. (2017). Mplus *(Version 8) [Computer software]*.

[B40-behavsci-16-01916] National Center for Education Statistics (n.d.). Concentration of public school students eligible for free or reduced-price lunch.

[B41-behavsci-16-01916] National Survey of Children’s Health (2017). 2011/12 NSCH stata indicator data set prepared by the data resource center for child and adolescent health, child and adolescent health measurement initiative.

[B42-behavsci-16-01916] Osborne M. C., Reidy D. E., Temple J. R., DeMello A., Lu Y. (2024). Examining the relation between early violence exposure and firearm-related experiences in emerging adulthood: A longitudinal cohort study. Psychological Reports.

[B43-behavsci-16-01916] Papachristos A. V., Wildeman C., Roberto E. (2015). Tragic, but not random: The social contagion of nonfatal gunshot injuries. Social Science & Medicine, Special Issue: Social Networks, Health and Mental Health.

[B44-behavsci-16-01916] Pardini D., Beardslee J., Docherty M., Schubert C., Mulvey E. (2021). Risk and protective factors for gun violence in male juvenile offenders. Journal of Clinical Child & Adolescent Psychology.

[B45-behavsci-16-01916] Phillips J., Springer F. (1992). Extended national youth sports program 1991–92 evaluation highlights, part two: Individual Protective Factors Index (IPFI) and risk assessment study *(Unpublished report prepared for the National Collegiate Athletic Association)*.

[B46-behavsci-16-01916] Prinz R. J., Foster S., Kent R. N., O’Leary K. D. (1979). Multivariate assessment of conflict in distressed and nondistressed mother-adolescent dyads. Journal of Applied Behavior Analysis.

[B47-behavsci-16-01916] Pulavarthi T. S., Fabio A., Miller E., Culyba A. J. (2024). Examining associations between school connectedness, social support, violence, and firearm carrying. Journal of Interpersonal Violence.

[B48-behavsci-16-01916] Richters J. E., Martinez P. (1990). Checklist of children’s distress symptoms: Self-report version.

[B49-behavsci-16-01916] Richters J. E., Saltzman W. (1990). Survey of children’s exposure to community violence: Parent report and self-report version.

[B50-behavsci-16-01916] Rothstein R. (2017). The color of law: A forgotten history of how our government segregated America.

[B51-behavsci-16-01916] Rowan Z. R., Schubert C. A., Loughran T. A., Mulvey E. P., Pardini D. A. (2019). Proximal predictors of gun violence among adolescent males involved in crime. Law and Human Behavior.

[B52-behavsci-16-01916] Ruback R. B., Shaffer J. N., Clark V. A. (2011). Easy access to firearms: Juveniles’ risks for violent offending and violent victimization. Journal of Interpersonal Violence.

[B53-behavsci-16-01916] Sadatsafavi H., Sachs N. A., Shepley M. M., Kondo M. C., Barankevich R. A. (2022). Vacant lot remediation and firearm violence—A meta-analysis and benefit-to-cost evaluation. Landscape and Urban Planning.

[B54-behavsci-16-01916] Sampson R. J., Raudenbush S. W., Earls F. (1997). Neighborhoods and violent crime: A multilevel study of collective efficacy. Science.

[B55-behavsci-16-01916] Schmidt C. J., Rupp L., Pizarro J. M., Lee D. B., Branas C. C., Zimmerman M. A. (2019). Risk and protective factors related to youth firearm violence: A scoping review and directions for future research. Journal of Behavioral Medicine.

[B56-behavsci-16-01916] Smith P. N., Cordell C., Stevens L. T., West K., Morgan S. T., Vallas J., Mehari K. R. (2025). Project GRIP: An illustration of participatory action research with communities of people who own and use firearms. Psychological Reports.

[B57-behavsci-16-01916] Stoddard S. A., Henly S. J., Sieving R. E., Bolland J. (2011). Social connections, trajectories of hopelessness, and serious violence in impoverished urban youth. Journal of Youth and Adolescence.

[B58-behavsci-16-01916] Sullivan T. N., Walsh C. S., Leslie C. E., Brown K. J., Thomson N. D. (2025). Chapter 17—Community-based approaches to prevent firearm violence. Handbook of gun violence.

[B59-behavsci-16-01916] Sumner S. A., Maenner M. J., Socias C. M., Mercy J. A., Silverman P., Medinilla S. P., Martin S. S., Xu L., Hillis S. D. (2016). Sentinel events preceding youth firearm violence. American Journal of Preventive Medicine.

[B60-behavsci-16-01916] Thompson V. M., Watson-Thompson J., Bernard D. L., Bah L. (2026). Addressing structural factors to prevent youth violence through a multisector collaborative approach. Prevention Science.

[B61-behavsci-16-01916] Towers S., Gomez-Lievano A., Khan M., Mubayi A., Castillo-Chavez C. (2015). Contagion in mass killings and school shootings. PLoS ONE.

[B62-behavsci-16-01916] Tracy B. M., Smith R. N., Miller K., Clayton E., Bailey K., Gerrin C., Eversley-Kelso T., Carney D., MacNew H. (2019). Community distress predicts youth gun violence. Journal of Pediatric Surgery.

[B63-behavsci-16-01916] Tremblay R. E., Nagin D. S., Séguin J. R., Zoccolillo M., Zelazo P. D., Boivin M., Pérusse D., Japel C. (2004). Physical aggression during early childhood: Trajectories and predictors. Pediatrics.

[B64-behavsci-16-01916] Zeman J., Shipman K., Penza-Clyve S. (2001). Development and initial validation of the children’s sadness management scale. Journal of Nonverbal Behavior.

