# Supplementary material for: Social-Ecological Predictors of Violent Firearm Use Among Youth
Source: Behav Sci (Basel). 2026 Oct 16;16(10):1916. doi: 10.3390/bs16101916 (PMC13701983; doi:10.3390/bs16101916)
Supplement: Supplementary file 1 [file behavsci-16-01916-s001.zip › behavsci-4389338-supplementary.pdf]

Supplemental Table for

Social-Ecological Predictors of Violent Firearm Use Among Youth

Supplementary Table S1

Correlations Among Variables

Variable	1	2	3	4	5	6	7	8	9	10	11	12	13	14	15	16	17	18
1. Male sex	—																	
2. Age	.02	—																
3. High poverty school	.01	**.17**	—															
4. White race [a]	-.03	**-.14**	**-.59**	—														
5. Other race [a]	.02	.01	-.06	**-.31**	—													
6. Sexual and/or gender minority	**-.24**	**.07**	.05	-.05	.06	—												
7. Media exposure to violence and guns	-.02	.03	**.10**	**-.13**	.01	**.09**	—											
8. Exposure to community violence via witnessing [b]	.01	.02	**.25**	**-.18**	.05	**.11**	**.33**	—										
9. Neighborhood safety and cohesion	.03	**-.10**	**-.25**	**.21**	-.03	-.06	-.05	**-.12**	—									
10. Presence of a caring adult - Positive items	-.01	**-.12**	**-.14**	**.11**	**-.06**	**-.13**	.03	**-.13**	**.27**	—								
11. Presence of a caring adult - Negative items	-.05	.06	**.19**	**-.17**	.01	**.08**	.01	**.09**	**-.21**	**-.27**	—							
12. School Safety Problems - Violence and Guns	-.05	**.09**	**.45**	**-.34**	.01	.05	**.07**	**.19**	**-.14**	**-.09**	**.19**	—						
13. School Safety Problems - Teacher Behavior	**-.11**	**.06**	**.25**	**-.18**	.02	**.09**	**.10**	**.18**	**-.11**	**-.09**	**.14**	**.67**	—					
14. School Climate	**.13**	-.07	**-.10**	.03	-.03	**-.08**	**-.10**	**-.16**	**.32**	**.29**	**-.12**	-.03	**-.18**	—				
15. Number of adverse childhood experiences	**-.13**	.07	.02	**-.07**	**.08**	**.21**	**.27**	**.31**	**-.20**	**-.20**	**.16**	**.07**	**.15**	**-.18**	—			
16. Close family member committed a violent crime	-.05	.01	**.09**	**-.11**	-.03	**.11**	**.24**	**.29**	**-.11**	**-.10**	**.09**	**.08**	**.10**	**-.09**	**.38**	—		

17. Close family member injured or killed due to violence	**-.08**	.03	**.18**	**-.20**	.03	**.11**	**.30**	**.29**	**-.15**	**-.07**	**.12**	**.16**	**.13**	**-.11**	**.42**	**.40**	—	
18. Family conflict	**-.15**	.05	.01	-.03	**.09**	**.18**	**.17**	**.20**	**-.19**	**-.45**	**.33**	**.10**	**.17**	**-.23**	**.37**	**.24**	**.18**	—
19. Household gun possession	-.04	**-.08**	**-.18**	**.20**	.02	.06	**.21**	**.14**	**.09**	**.08**	-.05	**-.19**	**-.09**	-.03	**.20**	**.12**	**.13**	**.12**
20. Lives with biological father	**.07**	**-.10**	**-.32**	**.31**	-.01	**-.09**	**-.10**	**-.15**	**.16**	**.13**	**-.13**	**-.19**	**-.13**	**.08**	**-.31**	**-.14**	**-.17**	**-.08**
21. Friend committed a violent crime	-.01	**.07**	**.14**	**-.13**	.04	**.07**	**.20**	**.31**	**-.11**	**-.13**	**.11**	**.12**	**.09**	**-.13**	**.27**	**.28**	**.24**	**.15**
22. Friend injured or killed due to violence	.01	**.08**	**.12**	**-.12**	**.06**	**.08**	**.27**	**.30**	-.06	**-.12**	**.10**	**.08**	**.11**	**-.11**	**.30**	**.26**	**.36**	**.16**
23. Friend carried a gun	**.10**	**.07**	**.08**	-.07	.03	.02	**.18**	**.26**	**-.08**	**-.07**	**.07**	**.07**	.06	**-.09**	**.20**	**.17**	**.20**	**.08**
24. Friends' delinquent behavior [b]	-.06	**.10**	.01	.02	.06	**.13**	**.27**	**.35**	**-.08**	**-.14**	**.11**	**.07**	**.11**	**-.20**	**.35**	**.25**	**.24**	**.23**
25. Substance use [b]	**-.09**	**.14**	.06	-.01	.07	**.17**	**.21**	**.46**	**-.10**	**-.19**	**.13**	**.09**	**.06**	**-.19**	**.30**	**.23**	**.23**	**.25**
26. Physical aggression [b]	.00	-.04	**.14**	**-.16**	**.09**	**.12**	**.32**	**.55**	**-.11**	**-.12**	**.13**	**.15**	**.13**	**-.17**	**.28**	**.24**	**.27**	**.25**
27. Physical violence [b]	-.03	-.05	.03	-.03	.04	**.15**	**.23**	**.54**	**-.13**	**-.15**	**.13**	**.12**	**.14**	**-.16**	**.32**	**.24**	**.27**	**.29**
28. Firearm-related victimization	.01	**.13**	**.36**	**-.27**	**.07**	**.16**	**.22**	**.49**	**-.15**	**-.22**	**.13**	**.18**	**.14**	**-.21**	**.29**	**.28**	**.27**	**.22**
29. Traumatic stress	**-.31**	.02	.01	.03	.06	**.27**	**.34**	**.32**	**-.11**	**-.11**	**.24**	**.08**	**.19**	**-.13**	**.44**	**.26**	**.30**	**.38**
30. Dysregulated anger expression	**-.15**	**.06**	.05	.03	.06	**.07**	**.12**	**.25**	-.04	**-.17**	**.11**	**.10**	**.07**	**-.09**	**.21**	**.13**	**.13**	**.26**
31. Positive outlook	**.11**	.02	**.15**	**-.16**	**-.07**	**-.28**	**-.07**	**-.10**	**.17**	**.30**	**-.22**	**.08**	-.04	**.23**	**-.32**	**-.13**	**-.11**	**-.32**
32. Beliefs about guns - Defensive escalation	**.10**	-.05	-.06	**.08**	-.02	.01	**.19**	**.13**	**.08**	.07	-.01	**-.09**	-.03	.02	**.08**	**.10**	**.08**	.05
33. Beliefs about guns - Compensatory force	.05	-.06	.01	-.02	.01	-.03	**.12**	**.11**	**.07**	.03	.03	-.03	**-.07**	.05	.05	**.08**	.03	.00
34. Beliefs about guns - Respect enforcement	**.07**	.05	**.24**	**-.24**	.01	-.02	**.08**	**.23**	**-.08**	**-.15**	**.14**	**.18**	.06	-.05	**.14**	**.13**	**.13**	**.11**
35. Reasons for storage - Self-defense	**.10**	**-.13**	**-.20**	**.16**	-.01	**-.07**	**.18**	.03	**.16**	**.26**	**-.06**	**-.16**	**-.08**	**.08**	.02	**.08**	.02	-.04
36. Reasons for storage - Inherent danger	.01	**-.12**	**-.23**	**.21**	-.05	.01	**.14**	-.05	**.18**	**.28**	**-.12**	**-.17**	**-.08**	**.14**	.01	.01	-.05	-.05

37. Negative gun attitudes	**-.25**	-.01	**.08**	**-.17**	-.01	**.23**	**.08**	.03	**-.07**	.05	.05	**.06**	**.08**	.03	**.11**	.03	.00	**.08**
38. Positive gun attitudes	**.20**	**-.09**	**-.25**	**.24**	.00	**-.16**	**.13**	.01	**.15**	**.12**	**-.12**	**-.21**	**-.17**	**.11**	-.02	.05	-.04	-.05
39. Firearm-related violence perpetration	**.10**	**.14**	**.24**	**-.16**	.02	.01	**.11**	**.26**	**-.12**	**-.12**	**.14**	**.12**	**.08**	**-.09**	**.15**	**.11**	**.13**	**.07**

Note. Significant correlations ($p < .05$) are indicated with bold text. [a] Reference group = Black race. [b] Variable was log-transformed.

Supplementary Table S1

Correlations among Study Variables (continued)

Variable	19	20	21	22	23	24	25	26	27	28	29	30	31	32	33	34	35	36	37	38
19. Household gun possession	—																			
20. Lives with biological father	**.10**	—																		
21. Friend committed a violent crime	**.09**	**-.08**	—																	
22. Friend injured or killed due to violence	**.10**	**-.12**	**.31**	—																
23. Friend carried a gun	**.10**	-.01	**.35**	**.23**	—															
24. Friends' delinquent behavior [a]	**.14**	**-.06**	**.37**	**.26**	**.54**	—														
25. Substance use [a]	**.15**	**-.09**	**.29**	**.23**	**.33**	**.53**	—													
26. Physical aggression [a]	**.15**	**-.13**	**.27**	**.25**	**.26**	**.39**	**.55**	—												
27. Physical victimization [a]	**.16**	-.06	**.27**	**.27**	**.26**	**.41**	**.48**	**.61**	—											
28. Firearm-related victimization	**.11**	**-.17**	**.27**	**.30**	**.27**	**.31**	**.35**	**.40**	**.38**	—										
29. Traumatic stress	**.18**	**-.13**	**.22**	**.22**	**.11**	**.28**	**.32**	**.27**	**.37**	**.23**	—									
30. Dysregulated anger expression	**.09**	**-.09**	**.17**	**.11**	**.12**	**.30**	**.34**	**.34**	**.25**	**.16**	**.27**	—								
31. Positive outlook	**-.09**	.02	**-.12**	**-.12**	-.06	**-.20**	**-.18**	**-.10**	**-.20**	**-.09**	**-.39**	**-.17**	—							
32. Beliefs about guns - Defensive escalation	**.30**	.04	**.09**	.05	.06	**.08**	.03	**.08**	**.12**	.03	**.13**	.01	-.05	—						
33. Beliefs about guns - Compensatory force	**.20**	.01	**.08**	**.07**	**.08**	**.08**	.06	**.12**	**.10**	**.10**	**.08**	.05	-.03	**.50**	—					
34. Beliefs about guns - Respect enforcement	.02	**-.11**	**.21**	**.11**	**.17**	**.19**	**.20**	**.23**	**.16**	**.27**	.05	**.16**	.00	**.28**	**.50**	—				
35. Reasons for storage - Self-defense	**.35**	**.08**	-.01	.01	-.01	.00	-.05	.02	.04	**-.11**	**.11**	-.06	.01	**.53**	**.35**	-.04	—			

36. Reasons for storage - Inherent danger	**.30**	**.11**	-.05	**-.07**	**-.08**	**-.09**	**-.12**	**-.11**	-.01	**-.17**	**.12**	**-.10**	.00	**.40**	**.20**	**-.15**	**.72**	—		
37. Negative gun attitudes	-.01	**-.15**	.00	.02	**-.11**	-.04	-.02	-.03	.05	.01	**.24**	-.05	**-.11**	.04	**.08**	.02	.06	**.19**	—	
38. Positive gun attitudes	**.32**	**.12**	.02	-.03	**.06**	.05	.02	.06	.05	-.02	-.04	.03	.05	**.42**	**.35**	**.12**	**.53**	**.35**	**-.18**	—
39. Firearm-related violence perpetration	.02	**-.09**	**.25**	**.18**	**.18**	**.20**	**.25**	**.19**	**.15**	**.34**	**.10**	.07	-.05	.02	**.10**	**.22**	**-.10**	**-.17**	-.04	-.01

Note. Significant correlations ($p < .05$) are indicated with bold text. [a] Variable was log-transformed.

Supplementary Table S2

Full Multivariate Model with All Hypothesized Social-Ecological Predictors of Youths' Violent Firearm Use

Measure	χ^2	*b*	***AOR*** **(95% CI)**
Demographics	25.41***		
Male sex		.72**	2.06 (1.23-3.44)
Age		.19	1.21 (.98-1.50)
High-poverty school		.77*	2.17 (1.00-4.68)
White race [a]		-.28	.76 (0.31-1.89)
Other race [a]		-.21	.81 (0.41-1.61)
Sexual and/or gender minority		-.37	.69 (0.33-1.45)
Cultural level	1.46		
Media exposure to violence and guns [b]		.16	1.17 (.91-1.52)
Community level	6.54		
Exposure to community violence via witnessing [b]		.17	1.19 (.93-1.52)
Neighborhood safety and cohesion [b]		-.15	.86 (.68-1.09)
Presence of a caring adult - Positive items [b]		.07	1.07 (.82-1.38)
Presence of a caring adult - Negative items [b]		.22	1.25 (.97-1.61)
School level	.42		
School Safety Problems - Violence and Guns [b]		-.03	.97 (.69-1.36)
School Safety Problems - Teacher Behavior [b]		.09	1.09 (.81-1.49)
School Climate [b]		.00	1.00 (.79-1.27)
Family level	3.16		
Number of adverse childhood experiences		.06	1.07 (.93-1.22)
Close family member committed a violent crime		-.36	.70 (.42-1.16)
Close family member injured or killed due to violence		-.12	.88 (.51-1.53)
Family conflict [b]		-.05	.95 (.72-1.26)
Household gun possession		.07	1.08 (.62-1.87)
Lives with biological father		.19	1.21 (.73-2.01)
Peer level	8.49		
Friend committed a violent crime		.73*	2.07 (1.17-3.68)
Friend injured or killed due to violence		.29	1.34 (.83-2.16)
Friend carried a gun		-.18	.84 (.47-1.50)

Friends' delinquent behavior [c]		.03	1.03 (.81-1.30)
<u>Intra-individual level – Problem behavior</u>	22.90***		
Substance use [b]		.53***	1.70 (1.29-2.23)
Physical aggression [b]		-.13	.88 (.66-1.18)
Physical victimization [b]		-.22	.81 (.62-1.04)
Firearm victimization		1.01***	2.76 (1.49-5.11)
<u>Intra-individual level – Mental health</u>	1.98		
Traumatic stress [b]		.11	1.11 (.83-1.48)
Dysregulated anger expression [b]		-.16	.85 (.64-1.12)
Positive outlook [b]		-.05	.95 (.72-1.25)
<u>Intra-individual level – Beliefs and attitudes</u>	17.74*		
Beliefs about Firearms - Defensive escalation [b]		-.02	.98 (.68-1.41)
Beliefs about Firearms - Compensatory force [b]		.35	1.42 (.95-2.12)
Beliefs about Firearms - Respect enforcement [b]		.14	1.15 (.86-1.53)
Reasons for storage - Self-defense [b]		-.11	.90 (.60-1.33)
Reasons for storage - Inherent danger [b]		-.36*	.70 (.50-0.99)
Negative Attitudes Toward Firearms [b]		-.09	.91 (.71-1.17)
Positive Attitudes Toward Firearms [b]		.06	1.07 (.79-1.44)

Note. χ^2 is provided by domain, and *b* is provided for single factors. AOR = Adjusted Odds Ratio. CI = Confidence Interval. [a] Reference group = Black race.[b] Variable was standardized in bivariate regression analysis to facilitate interpretation.

**p* < .05. ***p* < .01. ****p* < .001.

Supplementary Table S3

Full Multivariate Model of Social-Ecological Predictors of Youths' Violent Firearm Use Among the Pilot Sample Only

Measure	χ^2	*b*	*AOR* (95% CI)
Demographics	11.81*		
Male sex		1.04*	2.83 (1.03-7.75)
Age		.04	1.04 (.69-1.57)
White race[a]		-5.20*	.01 (.00-.29)
Other race[a]		.11	1.12 (.31-4.03)
Cultural level	1.07		
Media exposure to violence and guns[b]		-.28	.76 (.45-1.28)
Community level	6.91		
Exposure to community violence via witnessing[c]		.16	1.17 (.81-1.71)
Neighborhood safety and cohesion[b]		-.22	.80 (.51-1.26)
Presence of a caring adult - Positive items[b]		-.15	.86 (.58-1.29)
Presence of a caring adult - Negative items[b]		.43*	1.54 (1.05-2.28)
School level	.88		
School Safety Problems - Violence and Guns[b]		.06	1.06 (.63-1.79)
School Safety Problems - Teacher Behavior[b]		.11	1.12 (.65-1.93)
School Climate[c]		-.11	.90 (.56-1.44)
Family level	5.54		
Number of adverse childhood experiences		-.02	.98 (.77-1.24)
Close family member committed a violent crime		-.32	.73 (.27-1.98)
Close family member injured or killed due to violence		.41	1.51 (.56-4.09)
Family conflict[b]		-.28	.75 (.49-1.17)
Lives with biological father		-.68	.51 (.21-1.22)
Peer level	6.18		
Friend committed a violent crime		.15	1.16 (.34-3.95)
Friend injured or killed due to violence		.83	2.28 (.98-5.32)

Friend carried a gun		.46	1.61 (.55-4.70)
Friends' delinquent behavior[b]		.04	1.04 (.62-1.76)
Intra-individual level - Problem behavior	20.20**		
Substance use[b]		.86***	2.36 (1.51-3.68)
Physical aggression[b]		.18	1.19 (.69-2.05)
Physical victimization[b]		-.80**	.45 (.25-.79)
Firearm-related victimization		.77*	2.16 (1.05-4.44)
Intra-individual level - Mental health	1.30		
Traumatic stress[b]		.32	1.38 (.79-2.41)
Dysregulated anger expression[b]		.02	1.02 (.69-1.50)
Intra-individual level – Beliefs and attitudes	11.30*		
Beliefs about Firearms – Compensatory force[b]		.92*	2.50 (1.25-5.00)
Beliefs about Firearms - Respect enforcement[c]		-.03	.98 (.59-1.62)
Reasons for storage – Self-defense[b]		-.12	.89 (.50-1.60)
Reasons for storage - Inherent danger[b]		-.69*	.50 (.29-.86)

Note. χ^2 is provided by domain, and *b* is provided for single factors. AOR = Adjusted Odds Ratio; CI = Confidence Interval. [a] Reference group = Black race. [b]Variable was standardized to facilitate interpretation.

*$p < .05$. **$p < .01$. ***$p < .001$.
